# Valuing Health Surveillance as an Information System: Interdisciplinary Insights

**DOI:** 10.3389/fpubh.2019.00138

**Published:** 2019-06-13

**Authors:** Nicolas Antoine-Moussiaux, Olivier Vandenberg, Zisis Kozlakidis, Cécile Aenishaenslin, Marisa Peyre, Mathieu Roche, Pascal Bonnet, André Ravel

**Affiliations:** ^1^Fundamental and Applied Research for Animals and Health (FARAH), University of Liège, Liege, Belgium; ^2^Research Centre on Environmental and Occupational Health, School of Public Health - Université Libre de Bruxelles, Brussels, Belgium; ^3^Division of Infection and Immunity, Faculty of Medical Sciences - University College London, London, United Kingdom; ^4^International Agency for Research on Cancer (IARC/WHO), Lyon, France; ^5^Groupe de Recherche en Épidémiologie des Zoonoses et Santé Publique, Faculté de Médecine Vétérinaire, Université de Montréal, Montreal, QC, Canada; ^6^Department of Epidemiology, Biostatistics and Occupational Health, McGill University, Montreal, QC, Canada; ^7^ASTRE, Univ. Montpellier, CIRAD, Inra, Montpellier, France; ^8^TETIS, Univ. Montpellier, AgroParisTech, CIRAD, CNRS, Irstea, Montpellier, France; ^9^Department Environments and Societies, CIRAD, Montpellier, France

**Keywords:** complexity, decision-making, data, value, network, trend, typology, framework

## Abstract

The economic evaluation of health surveillance systems and of health information is a methodological challenge, as for information systems in general. Main present threads are considering cost-effectiveness solutions, minimizing costs for a given technically required output, or cost-benefit analysis, balancing costs with economic benefits of duly informed public interventions. The latter option, following a linear command-and-control perspective, implies considering a main causal link between information, decision, action, and health benefits. Yet, valuing information, taking into account its nature and multiple sources, the modalities of its processing cycle, from production to diffusion, decentralized use and gradual building of a shared information capital, constitutes a promising challenge. This work proposes an interdisciplinary insight on the value of health surveillance to get a renewed theoretical framework integrating information and informatics theory and information economics. The reflection is based on a typological approach of value, basically distinguishing between use and non-use values. Through this structured discussion, the main idea is to expand the boundaries of surveillance evaluation, to focus on changes and trends, on the dynamic and networked structure of information systems, on the contribution of diverse data, and on the added value of combining qualitative and quantitative information. Distancing itself from the command-and-control model, this reflection considers the behavioral fundaments of many health risks, as well as the decentralized, progressive and deliberative dimension of decision-making in risk management. The framework also draws on lessons learnt from recent applications within and outside of health sector, as in surveillance of antimicrobial resistance, inter-laboratory networks, the use of big data or web sources, the diffusion of technological products and large-scale financial risks. Finally, the paper poses the bases to think the challenge of a workable approach to economic evaluation of health surveillance through a better understanding of health information value. It aims to avoid over-simplifying the range of health information benefits across society while keeping evaluation within the boundaries of what may be ascribed to the assessed information system.

## Introduction

The economic evaluation of health surveillance systems remains a methodological challenge, as it is for information systems in general. This challenge is tied to that of defining what makes the value of health information, be it generated inside or outside of public surveillance systems. Main threads in this regard are presently considering cost-effectiveness solutions, minimizing costs for a given technically required output, cost-utility, then depending on the way the latter concept is interpreted and operationalized, or cost-benefit analysis, balancing costs with economic benefits of duly informed public interventions. While cost-effectiveness solutions are avoiding the question of benefits and are subject to public choices to be made on other bases, the present cost-benefit applications display some shortfalls restricting their relevance in face of the diversity of health risks.

Indeed, following a linear command-and-control perspective inspired from epidemic control procedures, those methods imply considering a simplified link between information, decision, action, and health benefits that is rather mono-causal and short-term. By neglecting the complexity of health issues and governance, these approaches may not take a faithful account of a diversity of outcomes accruing from surveillance, and might disfavor those oriented toward risks calling for long-term processes for their control. Also, the realm of surveillance, rapidly evolving in a highly connected world, fosters new challenges about the evaluation of information, its quality and its overall value. Hence, valuing information, taking into account its nature and multiple sources, the modalities of its processing cycle, from production to diffusion and decentralized use or effects, as well as the gradual building of a shared information capital and information commons, constitutes a promising challenge. This work reflects on the value of health surveillance with an interdisciplinary insight. Hopefully, these thoughts will add the previous and current research and thoughts on surveillance evaluation, especially its economics evaluation.

## Overview of Approaches in Valuation of Surveillance and Health Information

The question of economic evaluation rests on the basic question of what makes the value of the object under scrutiny. As introduced, present approaches of economic evaluation of health surveillance mostly tie the value of the system to the output of the actions it has informed, i.e., to the epidemiological evolution of the health risk to control. A refined framework places this economic analysis within a stake of optimal allocation of resources, based on the substitutability between surveillance and mitigation costs (1, 2). Hence, the value of surveillance more precisely lies in the avoided control costs. Mobilizing a micro-economic reasoning based on the marginal analysis of a mitigation function, this framework is most useful to evaluate programs for which mitigation interventions are directly informed by a surveillance system. However, concrete applications of this optimization framework remain a challenge as data on these trade-offs (substitutability) between surveillance and mitigation are not yet available (3). The value of surveillance is then evaluated jointly to that of intervention, through the avoided losses, both in public and animal health (4, 5). Since optimal allocation is out of reach in the present state of knowledge, economic evaluation then aims at comparing alternatives *ex ante* or evaluating *ex post* whether a realized program delivered the expected value for money (6).

Different authors recognize the possibility for a surveillance system to generate value even in absence of intervention, which is then considered as *intangible benefits*, such as knowledge (1, 7). These *intangible benefits* have been mostly left unevaluated economically, due to methodological difficulties in including this in the overall evaluation methodology. This neglect may also be understood as a consequence of the minor role ascribed to those intangibles compared to the concrete action-derived benefits, which seems indeed justified in the above-mentioned epidemic control systems.

Besides surveillance evaluation, the value of health information has been explored within the so-called Value of Information (VoI) framework (8). The VoI framework considers the usefulness of information in *improving* the decisions they underpin. This framework's operationalization will then basically depend on the extent to which one can observe the quality of a decision, which is not obvious. Practically, the improvement considered is one of uncertainty reduction and the VoI framework will then be implemented through Bayesian networks. Hence, the value of an additional information will be tied to an overall change in the network of conditional probabilities, with the quality of the decision being estimated *ex ante* through the probabilistic summing of monetary outcomes. This thinking thus pertains to marginal analysis, considering the value of an additional unit of information to assess whether it still exceeds its marginal cost. It is mostly mobilized in the context of diagnostic procedures, considering improvement along a gradient of certainty or accuracy of a single measure or through the use of complementary information (8).

## Fundaments to Rethink Health Surveillance Value

### What Is Information? From Data to Decision-Making

Information economics as developed 20 years ago by Shapiro and Varian (9) consider information as “anything that is or can be digitized” and that has value for consumers. Recent frameworks for information system analysis propose a fundamental distinction between data, information and knowledge, or intelligence, then fuelling decision-making (10, 11). Data will be considered here as anything that can be measured or characterized qualitatively and expressed in a way that can be transmitted and processed. Data is thus the raw material to produce information, which has the basic property of being meaningful for a user. Once integrated in a set of information and logically analyzed by human thinking, this information contributes to the gradual building of knowledge. Knowledge—i.e., human-processed information—is what logically and ideally should drive decision-making in complex settings. However, in more simple sub-systems or through the experience of a system's functioning, systematic procedures may be decided and data or information may be directly bonded to a particular action along established processes (12). The latter systematization then corresponds to the translation of information and then knowledge into organization (13).

In health surveillance, in both public or animal health contexts, the data considered are often the simple occurrence or prevalence of a pathological condition, an infection or disease, assessed through validated diagnostic protocols. In this context working groups conducted by ECDC (European Center for Disease Prevention and Control) have been established in order to work on the data quality monitoring (14). In the even more particular context of highly contagious infectious diseases (possibly transboundary or exotic) and foodborne diseases, the information system will be rather restricted to a function of (early) warning system (such as the European Surveillance System -TESSy- or PulseNet, the US national laboratory network that connects foodborne illness cases to detect outbreaks), often entailing pre-defined intervention procedures as detailed in contingency plans (e.g., import bans, product recall,…). Other surveillance systems may be built in the prospect of confirming freedom from a defined infection. However, this presentation of surveillance as a provider of point-in-time information misses the peculiarity of surveillance, which lies in its continuity and is best exemplified in the case of endemic diseases surveillance (15).

**Figure 1:**
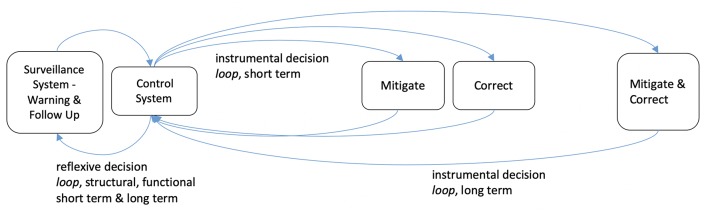
Health information uses along decisional loops.

Hence, surveillance data may be considered as generating two types of information: *punctual events* and *trends*. From the above, we may also propose to distinguish information value along a second dichotomy of functions, i.e., its *warning* or *follow-up* function. The decision that is pending on health surveillance may also be subject to a tentative typology. Obviously, the decisions may be directed toward the control of the health issue under scrutiny, which we may term *instrumental decision*s. From the two above-mentioned warning and follow-up functions of surveillance information, we may further derive two corresponding instrumental decision types, respectively, a mitigation decision (setting a time-bound control action) and a corrective decision (correcting the established control system). Other decisions may rather act on the surveillance system itself, here proposed as *reflexive decisions*, to be further distinguished between *structural* and *functional* changes. Indeed, while surveillance evaluation aims at such decisions, one should not overlook the role of surveillance output itself in deciding the increase or decrease of efforts in surveillance based on his satisfaction regarding the observed trends (16). Again, this points to the essential loop between information, decision and organization (13). The here-proposed dichotomy of information and ensuing decisions is summarized in Table 1 and Figure 1.

**Table 1:** Use values of health information considering decision type.

	**Punctual events**	**Trends**
Warning	Short-term instrumental decision: Mitigation	Long-term instrumental decision: Behavioral change Structural change
Follow-up	Short-term instrumental decision: Quality insurance Certification for freedom status	Long-term reflexive decision: Structural change Functional change
		Long-term instrumental decision: Corrective decision on control

### The Typology of Values Applied to Health Information

Stemming from philosophy and economics, different typologies of values are considered to better grasp the aims and methods of a valuation (17). Each domain of valuation will mobilize and further develop dichotomies in agreement with the nature of its object. For a detailed description of concepts and in-depth discussions, the reader may be redirected to works conducted in the domains of environmental valuation (17) and domestic biodiversity valuation (18).

First, a main case can be made here about the *instrumental* nature of the value of health surveillance, serving the ultimately valued objective of well-being, of human but also of animals, which increasingly appears as a value for its own sake. The latter precision pinpoints the fact that the opening of health issues to multiple disciplines and stakeholders within the One Health and EcoHealth approaches may require values at stake to be reconsidered, in a pluralist ethical perspective, connecting health to active debates in the two other domains cited here. Beyond that central instrumentality of health information, the present discussion may benefit from economic considerations distinguishing between use and non-use values. *Use value* is the value that agents ascribe to a good or service due to the use they have of the latter, while *non-use value* is derived by agents from the sole availability of the good or service, not involving any direct or indirect use of it. Use values may further be divided into active and passive use values, and *non-use values* into existence or bequest values. How these categories apply to health information will be later developed. An additional point to introduce among these fundaments is the economic notion of intrinsic value of an object, which we define here as the value an agent ascribes to an object *per se* [for detailed discussion of the various meanings of the term *intrinsic value* please refer to (17)]. This will invite us to clarify here the need to distinguish between the value of information, the value of the surveillance system and the way the two are related.

As considered in the VoI framework (8), the value of health information and surveillance will be directly determined by the context of uncertainty. This invites to a parallel with domestic biodiversity, for which uncertainty is described to foster two value types, i.e., the option and quasi-option values (18). The *option value* is tied to the recognition of potential, unknown future needs. It directly arises from risk aversion and holds clear relevance to health information. The *quasi-option value* is independent from risk aversion. It results from the irreversibility of decisions in biodiversity management and thus the need for information to improve that decision-making, motivating to conserve biodiversity until enough is known about future scenarios. Regarding health surveillance, this rhetoric of diversity and portfolio management may be also used to consider the array of data of interest that are collected about a set of health issues. Hence, one might consider a value to that diversity of surveillance objects as long as we lack the information needed to choose between health issues to be prioritized. This pinpoints the need to examine the value of a surveillance system within the set of surveillance systems in place, as far as the stake is one of allocation of limited resources between a diversity of risks. Also, for one defined health issue, the array of data to be collected may similarly display option and quasi-option values, since one may not be certain which data will be of relevance in the face of the evolution of the risk itself and our means to control it.

**Figure 2:**
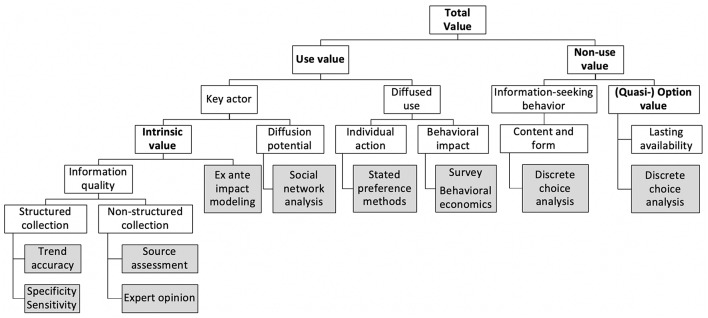
Tree linking value types (bold) with information characteristics (white) and valuation methods (gray). The distinction into use and non-use values leads to the inclusion of a diversity of tools of epidemiological or econometric nature. Each tool contributes to the modeling of a total value through the estimation of value-specific parameters.

For the following, we will consider that the value of health information may be instrumental in two ways. One is realized through the control of health risks and corresponds to its use value. The second responds directly to a behavioral need for information and may be equated to the non-use value of information, “use” being here restricted to the notion of decision-making. We propose here to first set out the relevance of considering non-use values and then to expose use values as a major area of methodological challenges. The next developments are summarized in Figure 2, which presents the proposed dichotomy of values together with economic valuation methods.

### Non-use Values

The direct value of information is driven by the aversion of actors to risk and to ambiguity, who will then express an information-seeking behavior, even in absence of pre-defined or resulting decisional stake. This information-seeking behavior is a growing field of research in health, mainly developed around doctors, patients or specific social categories [e.g., (19–22)]. Additional information may then provide a value to the user not by improving a decision or action but by directly responding to an ethological need for information. Let us note that the contrary may also be observed, valuable information not being sought for, or not enough, because of other psychological mechanisms, as denial or avoidance (23). The latter consideration leads us to consider the acceptability of information as an essential quality trait, adding value to it.

Much of this direct value of information will pertain to its form, i.e., the adequacy of the message expression with the targeted beneficiaries, easing its diffusion and understanding among actors as well as its acceptance. This conducts us to include the demand for information in the society at large within its valuation. This points to the crucial need for health information to diffuse beyond the sole technical services directly involved. Nevertheless, due to risks of panic, stigmatization, or economic losses caused by the reaction to information, diffusion may be questionable in some sensitive cases. These may even lead to disservices of health surveillance. Therefore, the choice of the content, formulation and medium for health messages and particularly of the treatment that is made of uncertainty in this communication will be of crucial importance in defining the value of information.

An information may be first recognized to have a non-use value for a defined actor but be retained till opportunities for use show up. Therefore, part of this non-use value may be also understood within the category of option value, also tied to risk aversion, or that of quasi-option value, that is still applicable to risk-neutral decision-makers. This would then indicate the persistence of the availability of information as well as the ease to organize it (repositories, interoperability) and memorize it, hence its simplicity, as a quality increasing this aspect of information value.

Defining the non-use value of information according to its meaningfulness to actors, the diversity of users entails a diversity of forms information has to take, a diversity of information and information services that has to be derived from a set of data, as well as a diversity of data that has to be gathered. Rather than starting from the epidemiological supply-side of health data and information, recognizing the non-use value of health information invites to jointly address the question from the demand-side. This sheds light on the diffusion function of surveillance system. Indeed, from the point of view of the whole surveillance system, the realization of this direct value entails the integration or close collaboration with health communication services, whose performance assessment will contribute to the valuation of surveillance. As for valuation methods, the demand for information may be assessed through well-established method of stated preferences, as provisionally tested by Delabouglise et al. (24) with Vietnamese farmers in the case of avian influenza. Such methods are widely used in raising values for environmental services or biodiversity (17, 18) and would benefit to surveillance evaluation by including a wider set of actors and citizens, then raising the social demand-derived value of health information.

### Use Values

#### Separating Value From Actual Consequences

In agreement with the introductory statements, it is important to consider the distinction between the use value of health information and the value of the decision and action through which it is mediated. This appears as the condition for a fair evaluation of health surveillance, i.e., preventing the confusion between a poor or delayed realization of actions and a poor surveillance performance or usefulness. Therefore, the present framework explicitly considers the use value as an intrinsic value of information or surveillance, which will then have to be handled and assessed on an *ex ante* basis. Hence the expected consequences of duly mobilized health information will be included in its evaluation, notwithstanding the reality of its concretization, based on theoretical models linking its characteristics to its usability. Such characteristics in fact mostly correspond to the presently established qualities of surveillance (6), which may then be distinguished between system-level features and information-level features.

#### Accounting for the Trend

To deal first with information-level value, a crucial yet neglected aspect of surveillance-produced information is its continuous nature, hence expressed through trends as mentioned earlier. Indeed, even when especially designed within the context of monitoring systems and continuous real-time surveillance [e.g., (25, 26)], present evaluation frameworks tend to neglect the time dimension of monitoring. Yet, the production of information about trends is what centrally distinguishes surveillance from any other point-in-time cross-sectional study. On another note, the latter might in fact generate a wider diversity or increased accuracy of information because it may be less limited for its simplicity and acceptability, two important features of surveillance systems. Therefore, the value derived from the continuity, time-points density and timeframe of datasets has to be acknowledged. These features of information intervene in trade-offs with other qualities and values and are subject to strategic and operational choices in active surveillance. In particular, dense and complete follow-up datasets will be needed to ascertain the absence of events, as opposed to an hypothesized one. This may be critical if the link between two or more outbreaks detected at different timepoints has to be interpreted, in absence or presence of data during the intervals.

Although the trend is a central value added of surveillance, its intrinsic value lacks a framework, for which we may propose the following basis. Let us consider a decision-maker facing follow-up information within its corrective and reflexive assignments, i.e., mobilizing health information in the continuous revision of actions or of surveillance itself. As a starting point, a surveillance system delivers to this decision-maker any of the three conclusions about the trend over a given period of time: increase, decrease or steady state. In face of this trend, the decision-maker can be satisfied or not, as dissatisfaction is a motivation for surveillance (16). Being satisfied, he/she will make the decision of maintaining the interventions and surveillance already in place. If one decision-maker is not satisfied with the trend, he/she certainly will make the decision to induce changes in the present risk management in order to modify the future trend in the satisfying way. He/she may decide implementing the interventions that were already planned or think over the most effective and efficient interventions. Unfortunately, as for any information, this trend may not correspond to the real evolution of the risk under surveillance. Thus, the true trend can be an increase, a decrease or a steady state, meaning that the conclusion of the surveillance can be wrong in six cases out of nine (Table 2). Whenever the decision to do something different or not is made on a wrong conclusion of the surveillance system in comparison to the actual trend, there is an economic cost. For example, not detecting a true increase will postpone the decision to act, hence paving the way for an increasing burden of the problem, whereas failing to detect the decreasing trend consecutive to a set of interventions will lead to the decision of maintaining, increasing or changing the interventions in place when they are in fact less or no longer relevant, hence unduly consuming resources.

**Table 2:** The decision-manager's satisfaction (S) in face of trend detected through surveillance, the decision (D) to do something different, the relevance of the decision (R) and the unnecessary economic cost incurred (UC) considering the true (unknown) trend.

		**True trend**		
		**Increase**	**Steady state**	**Decrease**
Estimated Trend	Increase	S: no D: yes R: yes UC: none	S: no D: yes R: no UC: unnecessary actions implemented	S: no D: yes R: no UC: unnecessary actions implemented
	Steady state	S: yes^*^ D: no R: no UC: increasing burden, expanding undetected	S: yes^*^ D: no (or yes) R: yes UC: none	S: yes^*^ D: no (or yes) R: yes (or no) UC: none
	Decrease	S: yes^**^ D: no^***^ R: no UC: increasing burden, expanding undetected	S: yes^**^ D: no^***^ R: yes UC: none	S: yes^**^ D: no^***^ R: no (or yes) UC: unnecessary actions maintained

S, satisfied with the detected trend yes or no; D, making decision to do something different (yes) or not (no) for controlling the issue and modifying the trend; R, relevance of the decision: yes if it fits the requirement according to the true trend no otherwise; UC, nature of the unnecessary cost incurred due to the decision to do or not to do something different relatively to the requirement by the true trend;

* or no if interventions already in place;

** especially if interventions in place;

*** *or yes to relax the interventions in place*.

The propensity of a system to produce conclusions about trends corresponding to the real evolution should thus be measured by the epidemiological sensitivity and specificity applied to trends. At present, three definitions of surveillance sensitivity exists: for case detection, for presence detection, and for outbreak detection (27). We believe the latter can usefully be expanded to deal with any trend in disease/agent occurrence as schematized in Table 2. A recent study assessing the statistical power of a surveillance system of endemic diseases in detecting trend revealed the great challenges of surveillance sensitivity related to trend (15). This should not impede our considerations for this capacity to detect medium to long-term trends, which is very specific to surveillance and crucial in raising awareness of any disease increase or in confirming the impact of interventions.

#### Data Diversity Within the Digital and Societal Evolutions of Surveillance

Health information within surveillance systems tends to be considered under a restricted understanding of diagnostic information regarding a well-defined condition and/or agent, as it was also conceived here above. Surveillance is then considered as an agent-specific tool. Yet, non-specific surveillance tends to gain importance, through the development of technical platform-based surveillance or syndromic surveillance in both public and animal health and the use of indirect indicators of health (25, 28–31). Structuring such surveillance systems, combining data of diverse natures, is a technical challenge *per se*, as also experienced e.g., in water quality surveillance (32), which will translate into an increased complexity of its valuation as a whole and of its components.

Also, less controlled information, which may provide important signals and be crucial in determining the success of health risk control, are not included in the evaluation since they do not fit into these well-specified performance assessment methodology. In this context, textual information on the web (e.g., news articles, official disease reports, newsletters, information flows on social networks) are relevant for early detection of emerging infectious disease outbreaks (33). For instance, Barboza et al. (34) have shown that the web monitoring systems can detect avian influenza epizootics 12.7 days before the official notification to the World Organization for Animal Health (OIE). Event-based biosurveillance systems have been created for mining articles published on the web (35). HealthMap (36) proposes an interactive map which uses word-processing algorithms from multiple sources like news feeds, expert accounts (e.g., ProMED-mail) and multinational monitoring reports, etc. To this we could add that direct exchanges between health surveillance agents and stakeholders may convey such crucial qualitative information about events, acts or motives, which may be critical in the correct interpretation of diagnostic data, though posing new methodological challenges for disambiguation and treatment of possible fake information. This remark may then further be extended to the management of rumors, which may enter fully into the scope of health surveillance, both in its functions of data collection and information diffusion.

In addition to the evolution in data sources and their accessibility, societal changes also influences the realm of health risk and the future of surveillance. Especially, global trade, mass gatherings and travel contribute to introducing pathogens into new populations, creating the potential to develop into epidemics and to generate data that relate to those outbreaks, e.g., West Nile virus introduction and spread in New York (37), post-festival measles outbreak in Germany (38) and H1N1 spread through airline transportation (39). Interestingly, in the above cases, even though the collection of data followed new routes, the interpretation was performed using the established public health approaches, showing a valuable degree of integration of unforeseen events inside the surveillance system.

Hence, the accrual of data relevant to surveillance also takes place outside of established structures. This may be handled by duly considering in a same framework the dichotomy between structured and unstructured collection of data, an attribute that might affect the quality, depth and timeliness of data collection, thus its intrinsic value. Outside of the healthcare realm, significant advances have been made in integrated systems, especially those relating to financial services (e.g., market behavior) (40) and primary production (e.g., agriculture) (41, 42) where the value creation of development of such a framework would be easier to document and quantify. In particular in the case of Chinese financial services, the accrual of both structured and unstructured data from multiple sources has had a beneficial impact in the provision of micro-credit to Small and Medium Enterprises (SMEs). Specifically in the latter case, low-income families and micro-enterprises in China lacked access to financial services not because they lacked creditworthiness but merely because banks and financial institutions historically lacked appropriate data, information and capabilities to access the creditworthiness of and effectively provide financial services to this financially disadvantaged group (43). Parallel developments in health would certainly deserve attention, representing new methodological challenges for valuation in which participatory ratings and expert opinions may hold an interesting role.

This decentralized collection of data and information further invites to adopt network thinking in the valuation. Hence, actors collecting data or information from different sources within networks should be identified and considered as sources for a centralized information management system (44). As a mirror of the willingness to pay for information, the willingness to accept compensation for delivering information may be also appreciated through stated preference methods (24). Where a diversity of data has to be gathered among a diversity of actors, collaborative systems may be imagined (45). The development of customizable platforms hosting data from different sources to enable precision medicine are a striking example. An economic evaluation should then adapt to such systems to include network and collaborative dynamics and take account of data or information of diverse quality, trust levels, sources and natures.

A strict exclusion of data and information from unofficial and uncontrolled sources may be done with an idea of reaching a technical optimum of data quality. However, such exclusion will be made at the expense of data diversity and will lower the vigilance regarding unexpected or not officially monitored events, referring to the option and quasi-option value of data diversity. The timeliness in detection of signals (e.g., infectious disease domain) is challenging due to the ever-growing amount of publications on the web and the heterogeneity of available data (i.e., social media, news, databases).

#### Informing a Decentralized Decision-Making

The frameworks presented in the introductory overview all consider surveillance under its function of informing centralized decision-making (policy-making, interventions), mainly in the public sector (46). In reality, health information is of interest to a wide range of users, both in the public and private sectors, including large or small organizations or even individuals and communities. Each use delivers a distinct contribution to the value to be recognized to information. If this decentralized use value may be considered in the context of decision-making, one should not downplay the even-more diffused behavioral impact of information. The latter thus constitutes a challenge to be valued on its own if fair evaluation of surveillance is to be built. Indeed, the range of benefits of surveillance includes awareness raising and change in attitudes and practices. While all these may be somehow considered as “actions” or “interventions”, these terms fail at translating the decentralized and plural nature of decision-making. Many efforts in economic and epidemiological modeling currently aim at representing the consequences of this decentralized decision-making [e.g., (47)]. Also the social dynamics behind behavioral change entail that information may be produced and diffused without any effect until the so-called “tipping point” is reached (48). Hence, the absence of consequences does not mean an absence of value of that information, which would be better judged through an evaluation of awareness in the population in relation to information access.

Recognizing the varying degree of decentralization of decision-making in the management of many health risks, this framework thus first calls practically for a typology of users and demands. Also unexpected users and uses have to be monitored. Since information is aimed at building knowledge and influencing practices, surveillance evaluation should assess knowledge, attitude and practices, analyzed in relation with the access to information. Stated preference methods and other participatory tools should contribute significantly to the evaluation of health policy from the point of view of its users, among others through the calculation and analysis of actors' willingness to pay (24, 49, 50). Efforts to further develop and insert those methods within evaluation frameworks should be pursued, as those would both produce meaningful values for information, and also distinguish points of potential improvements among the determinants of this value.

Networks effect in information diffusion and the role of particular actors within those networks would be accounted for in order to maximize the efficiency of diffusion (51). Indeed, some actors in networks show particular centrality and a high potential for information diffusion, or even further decryption and reformulation to reach a wider audience. Targeted diffusion and pushed information must be considered to reach these actors. Also, a special attention has to be devoted to the relationship to be built between the surveillance system and mass media, especially under crisis circumstances (52).

Finally, different users will operate at distinct scales, entailing different amplitudes of expected consequences from their use of information, hence distinct intrinsic values tied to their obtention of information. Some actors will eventually act at the international level, while most surveillance systems are designed and evaluated at the national level and might therefore miss these positive externalities in their valuation.

Hence methodological developments are needed to develop a demand-side valuation of health information, in agreement with the seminal works of Shapiro and Varian (9). Such methods may build on social network analysis and the modeling of decentralized decision-making, as well as health behavioral models. The modeling of intrinsic value of information based on detailed operations of a variety of agents with distinct amplitude in expected consequences represents a tremendous task, *inter alia* entailing challenges for the selection of relevant system boundaries, hence the range of stakeholders to be included (value chain actors, spatial territory inhabitants, social networks). However, such modeling would allow for optimizing strategies well beyond the sole checking of the value for money of surveillance programs. Also, this modeling might be restricted to key players only. For a wider set of smaller agents showing a use value of health information, one might consider using stated preference methods, as in the case of non-use value but targeting agents in their active role with respect to the health risk under consideration.

The outcomes considered above about a decentralized use of health information mainly pertain to changes in practices, the epidemiological effect of which (either positive or negative) would have to be modeled. Besides these health behavior effects, a variety of outcomes may ensue from the tactical or strategical use of information by actors, in social and economic spheres. Particularly relevant examples may be sought in the livestock sector, where animal health information appears as a strategic stake for economic players, would this be for a direct use, disclosure or withholding of this information (24, 44, 53). Directly referring to the structure of the sectors involved, the level of organization and empowerment in the chain (vertical and horizontal integration), these outcomes may then relate to equity and sustainability criteria that would be taken into account in the health surveillance valuation.

## Socio-Economic Features of the Value of Health Information

Beside the direct consequences for the development of new methods and tools for surveillance evaluation, the present framework also provides some insights on the socio-economic features of health information and surveillance, which may contribute to further methodological developments.

### Toward a Value Chain of Health Information

The processing of data into meaningful information, then aggregated to and analyzed within a body of workable knowledge, may be considered as a process adding value to a raw material in order to satisfy users' demand. Therefore, the application of value chain analysis framework may deliver interesting insights on surveillance. Value chain analysis is a common tool in economics, increasingly applied within the context of health risk management (53).

Just as any value chain analysis helps increasing the quality of the final product, improving governance, i.e., agreements between actors and the overall institutional framework in which those operate, its application to data and information could help evaluating socio-economic stakes along the involved network to improve its functioning. Equity concerns in value chain analysis, through the study of the distribution of the value-added between actors, also finds an interesting parallel in information networks. Hence, diverse contributors, supplying data, analysis or knowledge, should be acknowledged and find their remuneration in the system in order for it to be continuous and sustainable. These equity concerns then fully fall within the scope of an economic evaluation of surveillance.

### Management Consequences of Loops

The behavioral dimension of information seeking and the role of knowledge, thus of information, in driving this behavior leads to interesting loop effects. Such loop effects in the cross-determination between organization and information were already pinpointed in early works of information economics, referring to a complexity theory framework (13). According to the present proposition, information that a given stakeholder receives and integrates in his/her thinking will further influence his/her understanding, awareness of the unknown and raise new demands for information. Therefore, at first, stakeholders might be uncomfortable in asserting their needs for information, which will be formed, defined, and expressed in a progressive way, through an interactive process with the design and implementation of the information system (54). Hence, this process will translate into an increase of the demand and social value of information. This non-linear dynamic of information demand may justify initial active information-giving stages where the value of it cannot be directly derived from demand, rather expecting this demand to be later developed (i.e., information needs being supply induced). Later on, this non-linearity will invalidate the application of the law of diminishing returns to information production and diffusion. Therefore, an optimum would not be expected which would indicate the point at which informational efforts have to stop. Obviously, a particular actor (individual or institutional) may reach a level of information at which his capacity to manage this information get saturated (cognitive load), unless supported by algorithms. However, this saturation may lead to a decision to invest in the increase of this management capacity and better instruments for decision-making, rather than limiting surveillance effort. Hence, the decision of what to monitor. The decision of what to monitor and to what extent will remain a political decision, based on a prioritization that far exceeds the sole monetary or number-of-lives stakes, all the more in health where panic, public anxiety and political pressure may be determining. Socio-economics in this understanding will indicate how to do surveillance, not what to monitor or to what extent.

This same non-linearity dynamic leads to consequences on system's costs and sustainability. Indeed, from the growing awareness and demand for information may result a gradual sophistication of required information and potentially ever-growing expenses. However, progress of knowledge and understanding of health risk allows for gradual systematization of part of surveillance activities, freeing up resources for new needs. Hence, information systems are building on human and infrastructural capital but also on informational capital (a notion that is gaining popularity in business management but needs rigorous conceptualization). Also, actors' growing interest and participation may decrease the public cost of surveillance, through the expected decrease of willingness to accept compensation to contribute data or information. Crowd sourcing and private stakeholders networks would then be able to contribute to or generate efficient surveillance, documented in biodiversity surveillance (55). Finally, from the perspective of surveillance evaluation and information valuation, the priority should be given to adding value to the produced information from the perspective of contributing stakeholders, hence stimulating participation and therefore private and public contributions to the surveillance system in terms of time, financial and human resources.

## Applications of Particular Relevance

### A Framework Tailored for Complex Systems

The present reflections are all the more relevant for complex health risks, contrasting with the simple command-and-control approach that is still driving much of public decision-making in human and animal health. Indeed, the links between information, decision-making and action become harder to identify unequivocally in more complex surveillance systems, e.g., systems integrating a diversity of sources in non-agent-specific surveillance or systems in which direct interventions based on surveillance outcomes are not planned or operated by the same jurisdiction. Hence, taking account of this complexity in information management becomes all the more crucial as we engage in still poorly understood health risks, where much uncertainty and controversies are prevailing (56, 57). The surveillance of antimicrobial resistance at the human-animal interface is an example of surveillance system applied to such a complex issue. Many other relevant examples may be sought in endemic infectious diseases, behavioral health risks or environmental health, all of which require complex strategies for their control, involving a set of actors with distinct and sometimes conflicting decision-making processes. These socio-political barriers to the integration of risk assessment and governance will cause this complexity to remain (3), representing a lasting demand for surveillance evaluation taking this complexity into account.

### Antimicrobial Resistance: The Case of Canada

As mentioned earlier, antimicrobial resistance appears itself as a highly complex health risk involving various sectors, disciplines and actions in order to be controlled (58, 59). The surveillance of antimicrobial resistance, covering a wide range of pathogens, host species, environments and behaviors, may serve as a paradigmatic illustration of the present proposal. Indeed, these characteristics create an obvious distance with the mainstream economic evaluation of surveillance, as a system where information triggers control interventions in a linear logic. The Canadian Integrated Program for Antimicrobial Resistance Surveillance (CIPARS) stands as an example of a surveillance system whose impacts relies on its indirect influence on policies and behavior changes on the long term.

The CIPARS is managed within a national public health organization (Public Health Agency of Canada) and actively collects yearly resistance data on three critical microorganisms that are pathogens of humans (*Salmonella, Campylobacter*) or sentinel bacteria (*Escherichia coli*) from three main animal species (poultry, swine, bovine) at different points of the food chain, including the farm, the abattoir and retail food stores (60).

Participation of farmers and of slaughterhouses to this surveillance system is entirely voluntary, thus the quality of information produced by the system relies on a strong network of contributors whose contribution depends on the perceived usefulness of the system, which is realized at the value chain level. Feedback of information to this category of end users is then crucial for the sustainability and existence of the system. In addition, no direct public intervention is derived from the information that is produced over time. CIPARS aims at influencing decision-making indirectly, by raising awareness and influencing decision-making in a wide range of stakeholders. Indeed, CIPARS shares its information with more than 600 end users from interdisciplinary fields covering animal and public health, including livestock and poultry producers, veterinarians, physicians and licensing bodies, local, provincial and federal public health and animal health organizations, pharmaceutical organizations, drug and food regulators, animal and farm advocacy groups, and researchers. Capturing the value of such surveillance system crucially needs to widen the current barriers of surveillance evaluation (61).

### The Consolidated Microbiology Networks

The consolidated microbiology networks (CMNs) are an example of another complex health system. They are existing non-agent-specific infrastructures that constitute an emerging structure within healthcare systems, where the provision of diagnostic services is centralized for multiple healthcare providers into a single, new servicing unit (62). The emergence of CMNs has been made possible through the technological advances in the field of microbiology, allowing the simultaneous access to information and processes, in real-time, and by many system users; as well as the ability to multiplex technical components providing a highly automated and standardized laboratory. It is anticipated that in the future such CMNs would play an increasing role in the routine health surveillance networks and act as a major informational conduit, where well-defined and structured data will be collected, interpreted and distributed for downstream decision-making.

The creation and consolidation of CMNs is aided by the fact that data from microbiology laboratories differ from most other categories of patient data—blood pressure, body mass index, etc. In the latter categories data can often become interdependent requiring a higher informational load prior to taking a decision. Microbiological analysis on the other hand reports on specific, well-defined parameters on two independent living organisms, patient and microbe. Data can thus often be accessed and analyzed in useful ways independently of other healthcare data (63). This independence and interest *per se* of microbiological analyses does not preclude, however, the need to analyze those in relation with patient data, especially to assess the impact of infections (and crucially resistant pathogens) on health and burden of disease. This again stresses the diversity of stakes in surveillance data analysis, hence of the information to be generated.

CMNs could become the first step toward a global set of sensor networks for infectious diseases surveillance, where each one of the world's microbiology laboratories can be seen as a real-time sensor and an increasingly discriminating reporter of the microbes infecting patients in its area within an interconnected, complex network (64). This makes good clinical and practical sense. Most hospitals have infection preventionists, each of whom works intensively to monitor the infectious diseases trends, inform surveillance and control such spread within their own hospital but often with little knowledge of what is trending in the next hospital, or another part of the country. Comprehensive, globally interpreted data shared between different end users and health agencies is not technically impossible and might help to close this gap. In such a scenario though the pressure to establish a value framework beyond the existing approaches is likely to intensify.

## Conclusion

Quite obviously, the present thoughts do not pretend making of a complex matter a simple question or providing simple answers. Nor does it dismiss the usefulness of the present evaluation frameworks, which have been guided by a highly practical sense and the need to come up with values and fuel the public decision-making. However, facing complex health risks, as underlined by the One Health or EcoHealth frameworks, with highly decentralized drivers, information valuation and surveillance evaluation should gain insights from a broader account of socio-economic and information sciences. Due to technological advances, information represents a central challenge in today's world and will certainly continue to do so in the future. The tremendous change in data collection, volume, processing and diffusion abilities, and in the interface between individuals and information, calls health surveillance economic evaluation to get addressed within interdisciplinary teams, well beyond health professionals or economists.

## Author Contributions

AR, MP, and PB designed the theoretical ideas on which the work is based. NA-M wrote the first draft of the manuscript, coordinated the work, integrated co-authors' inputs. OV, ZK, CA, and MR wrote sections of the manuscript. All authors of the manuscript significantly contributed to the critical revision and enrichment of all the sections of the work, read and approved the submitted version.

### Conflict of Interest Statement

The authors declare that the research was conducted in the absence of any commercial or financial relationships that could be construed as a potential conflict of interest.
